# Getting Rid of Dichotomous Sex Estimations: Why Logistic Regression Should be Preferred Over Discriminant Function Analysis

**DOI:** 10.1111/1556-4029.14482

**Published:** 2020-06-10

**Authors:** Bjørn Peare Bartholdy, Elena Sandoval, Menno L. P. Hoogland, Sarah A. Schrader

**Affiliations:** ^1^ Faculty of Archeology Leiden University Einsteinweg 2 Leiden 2333 CC The Netherlands

**Keywords:** sex estimation, sexual dimorphism, anthropometrics, discriminant function, linear discriminant analysis, logistic regression, humerus

## Abstract

Sex estimation is an important part of creating a biological profile for skeletal remains in forensics. The commonly used methods for developing sex estimation equations are discriminant function analysis (DFA) and logistic regression (LogR). LogR equations provide a probability of the predicted sex, while DFA relies on cutoff points to segregate males and females, resulting in a rigid dichotomization of the sexes. This is problematic because sexual dimorphism exists along a continuum and there can be considerable overlap in trait expression between the sexes. In this study, we used humeral measurements to compare the performance of DFA and LogR and found them to be very similar under multiple conditions. The overall cross‐validated (leave‐one‐out) accuracy of DFA (75.76–95.14%) was slightly higher than LogR (75.76–93.82%) for simple and multiple variable equations, and also performed better under varying sample sizes (94.03% vs. 93.78%). Three of five DFA equations outperformed LogR under the *B* index, while all five LogR equations outperformed the DFA equations under the *Q* index. Both methods saw an improvement in overall accuracy (DFA: 86.74–95.79%; LogR: 86.74–95.76%) when individuals with a classification probability lower than 0.80 were excluded. Additionally, we propose a method for calculating additional cutoff points (PMarks) based on posterior probability values. In conclusion, we recommend using LogR over DFA due to the increased flexibility, robusticity, and benefits for future users of the statistical models; however, if DFA is preferred, use of the proposed PMarks facilitates future analysis while avoiding unnecessary dichotomization.

Discriminant function analysis (DFA) and logistic regression (LogR) are common statistical methods for estimating sex in both forensic (1, 2, 3, 4) and osteoarcheological contexts (3, 5, 6). Statistical models are built from reference samples, which can then be applied to future cases for sex estimation. It is important to have many different reference samples (i.e., anatomical collections and/or archaeological samples with known sex) given that the expression of sexually dimorphic traits varies between populations (7, 8). While there have been many sex estimation equations provided from a variety of populations to deal with the inter‐population differences in sexual dimorphism (2, 9, 10, 11), there are still issues when it comes to the biological overlap that occurs between the sexes within a population (12). One way to offset intrasexual variability is by providing estimates associated with a probability, which can readily be obtained from DFA and LogR; however, the ability to obtain probabilities for predictions does not always carry over from the original publication to future users of the model. This is especially the case for DFA, where future users of the model are often only provided with an equation and a cutoff point (a point equidistant between the sexes) from the original publication, which results in a dichotomization of sex estimation. In reality, there should be multiple categories to reflect the uncertainty associated with the estimate. In this study, we explore the efficacy of DFA and LogR for building adult sex estimation models and provide recommendations for publishing results of DFA research in the future.

## Discriminant Function Analysis

Discriminant Function Analysis (DFA) is used to predict group membership from a single variable or multiple predictor variables, and/or describe the relationship between a predictor variable(s) and a criterion variable (groups). One way this is done by using linear discriminant funtions (LDF), straight lines that achieve maximum separation of the groups by passing through the group centroids; this may also be referred to as Fisher’s linear discriminant analysis (LDA). The equation for the straight line is determined and used to predict group membership. The discriminating line may also be nonlinear, for example, quadratic discriminant analysis (QDA). The boundary that separates the two groups is often represented as a cutoff point (a.k.a. demarking point, decision point, or sectioning point). The number of LDFs needed for the analysis depends on the number of groups or variables in the analysis and is defined as the smallest number of *k*−1 and *p*, where *k* is the number of groups and *p* is the number of variables; thus, in sex estimation studies there is only one equation to discriminate between male and female individuals, using a line that is equidistant between the two group means (or centroids). The assumptions of LDA can be found in Table 1 (13). The discriminant score is obtained and whichever side of zero it lands on indicates the predicted sex (e.g., female < 0 < male). The equation for calculating the linear discriminant score (*D*) is the following:

$$D=b_{0}+b_{1}x_{1}+b_{2}x_{2}+\ldots +b_{n}x_{n}$$

where *b*_0_ is the constant, *b_n_* is the unstandardized coefficients, and *x_n_* is the variables. Posterior probabilities can be obtained using multiple methods which may differ depending on the statistical software used. The methods include Bayes’ theorem and the Mahalanobis distance.

**Table 1:** Assumptions of discriminant function analysis and logistic regression.

Model	Assumptions
Discriminant function analysis	Multivariate normality.
	Absence of outliers.
	Independence of errors (each response comes from independent case).
	Homogeneity of variance–covariance matrices within groups.
	Linearity of pairs of predictors within groups.
	Absence of multicollinearity among predictors.
Logistic regression	Absence of outliers.
	Independence of errors (each response comes from independent case).
	Linearity between logit of the outcome and predictor variables.
	Absence of multicollinearity among predictors.

## Logistic Regression

LogR relies on the same concept as linear regression; however, since it uses categorical variables, a transformation of the data is needed. This transformation, or link function, is a logarithmic transformation (logit) of the outcome variable, which allows the analysis to maintain linearity in nonlinear categorical data. When predicting membership of two groups, LogR is referred to as binomial logistic regression, and the results are expressed as the probability of group membership in the form of a value between zero and one. LogR also enables the user to examine the odds ratio (OR) for each predictor variable, which allows you to see the effect a certain variable has on the outcome, with an OR of 1 being no effect (i.e., chance alone), and anything above (as OR approaches infinity) or below 1 (as OR approaches 0) represents an increasing effect on the outcome. LogR uses maximum likelihood to calculate the coefficients of the equation, that is, a model is created that maximizes the probability of an outcome based on the data. The assumptions of LogR can be found in Table 1 (13, 14). The probability of membership to the group coded as 1 (*p*_1_) can be calculated as:

$$P_{1}=\frac{1}{1+e^{-\left(b_{0}+b_{1}x_{1}+b_{2}x_{2}+\ldots +b_{n}x_{n}\right)}}$$

where *b*_0_ is the intercept, *b_n_* is the coefficients, and *x_n_* is the variables. The probability obtained is the probability of assignment to the group that was coded as 1. The probability of assignment to the group coded 0 can be obtained with the probability of not being in group 1: *p*_0_ = 1−*p*_1_.

## The Problem

Forensic and osteoarcheological analyses require accurate sex estimation equations for identification of a body, or for other methods (e.g., stature and age at death) that often rely on a classification of sex for precision (15). The problem with DFA arises when the developed sex estimation equations are published. Often, the equation will be presented with an accompanying cutoff point, which allows users to discriminate between male and female individuals. When the measurement(s) is inserted into the equation, the result determines whether the individual is male or female based on which side of the cutoff point the result lands. This creates an illusion of certainty in a practice where uncertainty is inherent, and limits future users of the model by not providing them with a quantifiable reliability measure.

Classification probabilities for future unknown cases can be directly obtained from the LogR equations, and posterior probabilities can be calculated for DFA. This is a convenient solution to the problem of dichotomization, as it provides the ability to make an informed prediction. While LogR allows for convenient calculation of classification probabilities directly from the provided equation, DFA, however, relies on authors of the discriminant functions making their data available to calculate posterior probabilities. Hence, merely providing the DFA equation(s) and a cutoff point does not allow future users to properly quantify the uncertainty of the estimate, as these cutoff points merely represent an assignment probability of 0.50. This ultimately results in a dichotomous decision which is not suited for sex estimation of skeletal remains. Methods to calculate the reliability of an estimate (in the absence of posterior probabilities) have been proposed in previous studies (9, 16, 17), but have not experienced widespread implementation.

Our aim is to compare the performance of both DFA and LogR when applied to the same reference sample and show the benefits when sex estimates are associated with a probability and/or multiple categories rather than a simple male or female determination. We also provide a method to calculate more appropriate cutoff points for both simple and multiple variable DFA equations using bootstrapping. We use three humeral measurements to illustrate our point by determining the number of misclassifications occurring below a probability of 0.80, and the improved accuracy rate and reliability of a model when only considering sex classifications with a higher probability, thus emphasizing the benefit of LogR over DFA and the need for additional cutoff points and/or sharing of data.

## Materials and Methods

### Reference Sample

The sample is comprised of 84 individuals with documented age and sex from Middenbeemster, a 19th‐century rural site located in the Netherlands. The cemetery was in use from AD 1612 to 1866, and a cemetery ledger is available for individuals interred between 1829 and 1866, which includes name, sex, date of birth, and date of death. More information on the population can be found elsewhere (18, 19, 20). The sample consists of 48 (57.14%) females with mean age 48.73 years (SD = 18.51), and 36 (42.86%) males with mean age 52.89 years (SD = 21.01). The ages range from 19 to 84 years. All available adults with known sex and preserved humeri were included. The collection is curated at Leiden University, Laboratory for Human Osteoarcheology.

### Measurements

Three humeral measurements were taken: max length, head diameter, and epicondylar breadth (21). The second author (ES) performed all measurements without prior knowledge of the sex of each individual. In order to facilitate a direct comparison between all the developed models, we only include individuals with all three measurements. Measurements were taken on the right side. If a measurement was not available on the right side, the left side measurement was substituted.

### Assumption Tests

Assumptions were tested using a Shapiro–Wilk test for multivariate normality, Mahalanobis distances for outliers, a variance inflation factor (VIF) test for multicollinearity, and Box’s M test for homogeneity of covariance matrices. Linearity between logit of the outcome and predictor variables was tested by running a logistic regression with the interaction terms of the predictors and their log (22).

### Model Comparison

Both simple (one predictor variable) and multiple variable (2 + predictor variables) LogR and LDA models were developed. Variable selection has become important with the philosophy of transition analysis (23, 24), especially in explanatory research. We have chosen the conventional variable selection for LogR, that is, sex as the grouping variable and the measurements as the predictors, and to facilitate direct comparison between DFA and LogR. DFA employs canonical correlation, and therefore, the variables do not take on an independent–dependent designation.

Linear discriminant analysis (LDA) was conducted with an uninformative prior. Accuracy rates were obtained from both models using leave‐one‐out cross‐validation (LOOCV). The effect of sample size on accuracy for both methods was explored by taking random subsamples, with replacement, of increasing sizes (*n* = 10, 11, …, 84). The methods were also compared using the Brier score, *B* (25, 26), and the logarithmic probability scoring rule, *Q* (27). Both scores provide a predictive index that takes into account the prediction accuracy as well as the classification probability. In the case of both scales, a score of 1 indicates perfect predictive ability, and scores based on chance alone are indicated by 0; for the *Q* index, negative scores indicate worse than chance.

$$B=1-\frac{\sum_{i=1}^{n}{\left(P_{i}-Y_{i}\right)}^{2}}{n}$$

$$Q=\frac{\sum_{i=1}^{n}\left[1+\log_{2}\left(P_{i}^{Y_{i}}{\left(1-Pi\right)}^{1-Y_{i}}\right)\right]}{n}$$

### Calculation of Additional Cutoff Points

Additional cutoff points (referred to as PMarks hereafter) were calculated for LDA to represent a sex classification probability of 0.80 using a custom‐made function in R v. 3.5.2 (28). The sample was bootstrapped (1000 iterations), and the discriminant scores for individuals from each sex that had a 0.80 posterior probability were found, and the mean values for the discriminant scores were used as the PMarks. Here, we calculate 0.80 PMarks, but this can be customized to suit the user’s need. See https://github.com/bbartholdy/pmarkr for information on how to download the R package, pmarkr, used to calculate PMarks (and the R script for statistics conducted in this study). The R package also contains a user‐friendly interface for those who are unfamiliar with R.

### Statistics

All statistical analyses were conducted in R v. 3.5.2 (28). LDA was conducted using the *lda* function in the MASS package with the "plug‐in" method for computing posterior probability (29), and LogR was conducted using *glm* function in the stats package (28). Plots were created using ggplot2 (30). Assumptions were tested using the mvnorm package (31), the heplot package (32), and the mvoutlier package (33). Other packages used include doParallel (34) and foreach (35).

## Results

### Tests of Assumptions

The multivariate normality assumption holds for the male group, *W* = 0.9570, *p* = 0.1730, while it is violated for the female group, *W* = 0.8953, *p* < 0.001. Only four outliers were identified: three females (MB5, MB8, MB96) and one male (MB13). None of the outliers were removed from the models. No multicollinearity was detected for any of the variables: max length = 1.418; head diameter = 1.745, epicondylar breadth = 1.5863. Homogeneity of covariance matrices was not violated: χ^2^(3) = 7.0388, df = 3, *p* = 0.3173. Linearity between the logit of the outcome and the predictors was also met. Max length: *z* = −0.137, *p* = 0.8908; head diameter: *z* = 1.160, *p* = 0.2461; epicondylar breadth: *z* = −1.713, *p* = 0.0867.

### Linear Discriminant Analysis

The descriptive statistics for the humeral measurements from male and female individuals can be found in Table 2. The max length variable had the lowest cross‐validated accuracy of all the variables, and contributed nothing to the predictive accuracy (as seen by comparison of models LDF4 and LDF5); the variable was, therefore, excluded from the model. The model with the head diameter and epicondylar breadth variables (LDF4) and the model with all variables performed equally well, so LDF4 was chosen as the optimal model (Table 3).

$$D=0.3381\times \left[\text{head}\;\text{diameter}\right]+0.06205\times \left[\text{epicondylar}\;\text{breadth}\right]-19.07.$$

**Table 2:** Mean values for the humeral measurements of male and female individuals including the mean difference with 90% confidence intervals calculated using Welch’s two sample *t*‐test.

Measurement	Mean (mm)	SD	Mean Difference	90% CI
Max length	F: 311.8	19.49	27.65	20.53–34.77
	M: 339.4	19.33		
Head diameter	F: 41.62	2.312	7.726	6.773–8.678
	M: 49.34	2.781		
Epicondylar breadth	F: 55.31	3.282	8.441	6.883–10.00
	M: 63.75	4.819		

90% CI, 90% confidence intervals; SD, standard deviation.

**Table 3:** LDA results for simple and multiple variable models.

Model	Measurement(s)	Coefficients	CV Accuracy (%)
LDF1	Max length	0.05149	75.76
	(intercept)	–16.77	
LDF2	Head diameter	0.3963	92.71
	(intercept)	–17.81	
LDF3	Epicondylar breadth	0.2493	86.67
	(intercept)	–14.84	
LDF4	Head diameter	0.3382	95.14
	Epicondylar breadth	0.06205	
	(intercept)		
LDF5	Max length	−0.001785	95.14
	Head diameter	0.3431	
	Epicondylar breadth	0.06354	
	(intercept)	–18.58	

CV, cross‐validated.

**Fig. 1:**
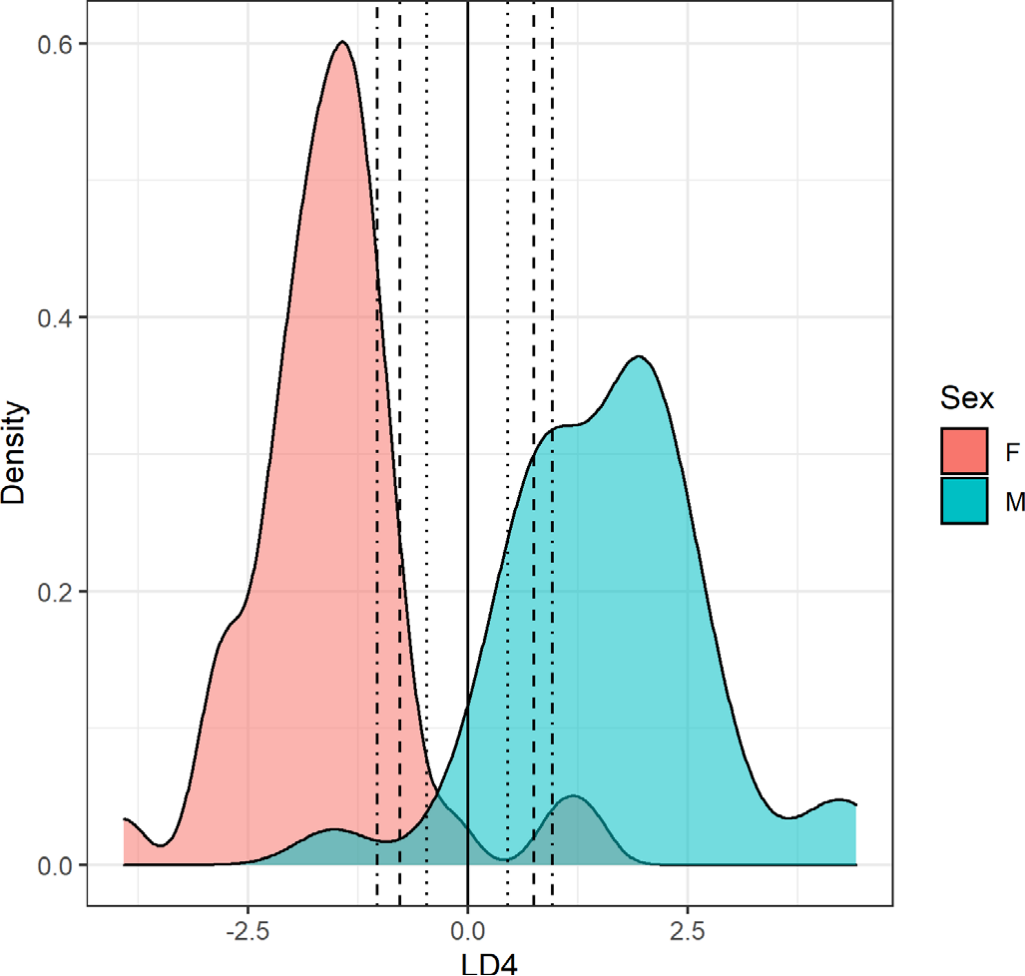
Density plot for LDF4. The straight line (‒‒‒) represents the traditional cutoff point; the dotted lines (∙∙∙), 0.80 PMarks; the dashed lines (‐‐‐), 0.90 PMarks; the dashed and dotted lines (∙‐∙‐∙), 0.95 PMarks. F, female; LD4, discriminant scores; M, male. [Color figure can be viewed at wileyonlinelibrary.com]

The traditional cutoff point for LDF4 is at 0 (Female < 0 < Male). The 0.80 PMarks are −0.45 for females and 0.45 for males. Scores in between these two values represent a posterior probability below 80% and should therefore be classified as either indeterminate, or probable male for values between 0 and 0.45, and probable female for values between 0 and −0.45. The 0.90 and 0.95 PMarks are ±0.76 and ±0.99, respectively. The posterior probability of all individuals can be found in Table S1. Figure 1 shows the plot for LDF4 and the calculated PMarks.

### Logistic Regression

The variable max length had an OR value close to 1 in model LogR5 and was excluded. The model containing the head diameter and epicondylar breadth variables (LogR4) was chosen as the optimal model, as it scored better in log‐likelihood (LogLik) and to facilitate comparison with LDA. LogR2 scored best in Akaike’s information criterion (AIC) and LogR2 and LogR4 performed equally well in cross‐validated accuracy (Tables 4 and 5). The probability of a female prediction can be calculated using the following equation:

$$P\left(\text{Female}\right)=\frac{1}{1+e^{-\left(43.42+\left(-0.8329\times \left[\text{head}\;\text{diameter}\right]\right)+\left(-0.09285\times \left[\text{epicondylar}\;\text{breadth}\right]\right)\right)}}$$

The probability of a male prediction can then be calculated as the probability of not being female: *P*(Male)=1 − *P*(Female).

**Table 4:** LogR results and model evaluation.

Model	Measurement	Coefficients	OR	LogLik	AIC
LogR1	Max length	−0.07356	0.9291	−40.54	85.07
	(intercept)	24.19			
LogR2	Head diameter	−0.9423	0.3897	−15.16	34.32
	(intercept)	42.88			
LogR3	Epicondylar breadth	−0.5220	0.5933	−27.77	59.54
	(intercept)	31.16			
LogR4	Head diameter	−0.8329	0.4348	−14.93	35.87
	Epicondylar breadth	−0.09285	0.9113		
	(intercept)	43.42			
LogR5	Max length	−0.003169	0.9968	−14.93	37.86
	Head diameter	−0.8254	0.4381		
	Epicondylar breadth	−0.09060	0.9134		
	(intercept)	43.97			

AIC, Akaike information criterion; LogLik, log‐likelihood; OR, odds ratio.

**Table 5:** LDA and LogR models with cross‐validated accuracy.

Model	Decision Probability (cutoff)	Misclassifications (total)	CV Accuracy (%)	Female (%)	Male (%)
LDF1	0.5 (0)	20 (84)	76.19	78.00	73.53
	0.8 (± 0.97)	4 (35)	88.57	91.67	81.82
LDF2	0.5 (0)	6 (84)	92.86	93.75	91.67
	0.8 (± 0.46)	3 (78)	**96.15**	**97.83**	93.75
LDF3	0.5 (0)	12 (84)	85.71	83.33	**90.00**
	0.8 (± 0.70)	3 (63)	**95.24**	**94.87**	95.83
LDF4	0.5 (0)	4 (84)	**95.24**	**95.83**	**94.44**
	0.8 (± 0.45)	3 (78)	**96.15**	**97.83**	93.75
LDF5	0.5 (0)	4 (84)	**95.24**	**95.83**	**94.44**
	0.8 (± 0.45)	3 (78)	**96.15**	**97.83**	93.75
LogR1	0.5	20 (84)	76.19	78.00	73.53
	0.8	4 (35)	88.57	91.67	81.82
LogR2	0.5	5 (84)	**94.05**	**95.74**	**91.89**
	0.8	3 (77)	96.10	97.78	93.75
LogR3	0.5	11 (84)	**86.90**	**87.76**	85.71
	0.8	3 (56)	94.64	93.75	95.83
LogR4	0.5	5 (84)	94.05	95.74	91.89
	0.8	3 (77)	96.10	97.78	93.75
LogR5	0.5	5 (84)	94.05	95.74	91.89
	0.8	3 (77)	96.10	97.78	93.75

Decision probability represents the probability level (between 0 and 1) at which the male and female assignment was made, and the cutoff is the discriminant score associated with the probability level. Bold indicates the model that performed best.

### Model Comparison

**Fig. 2:**
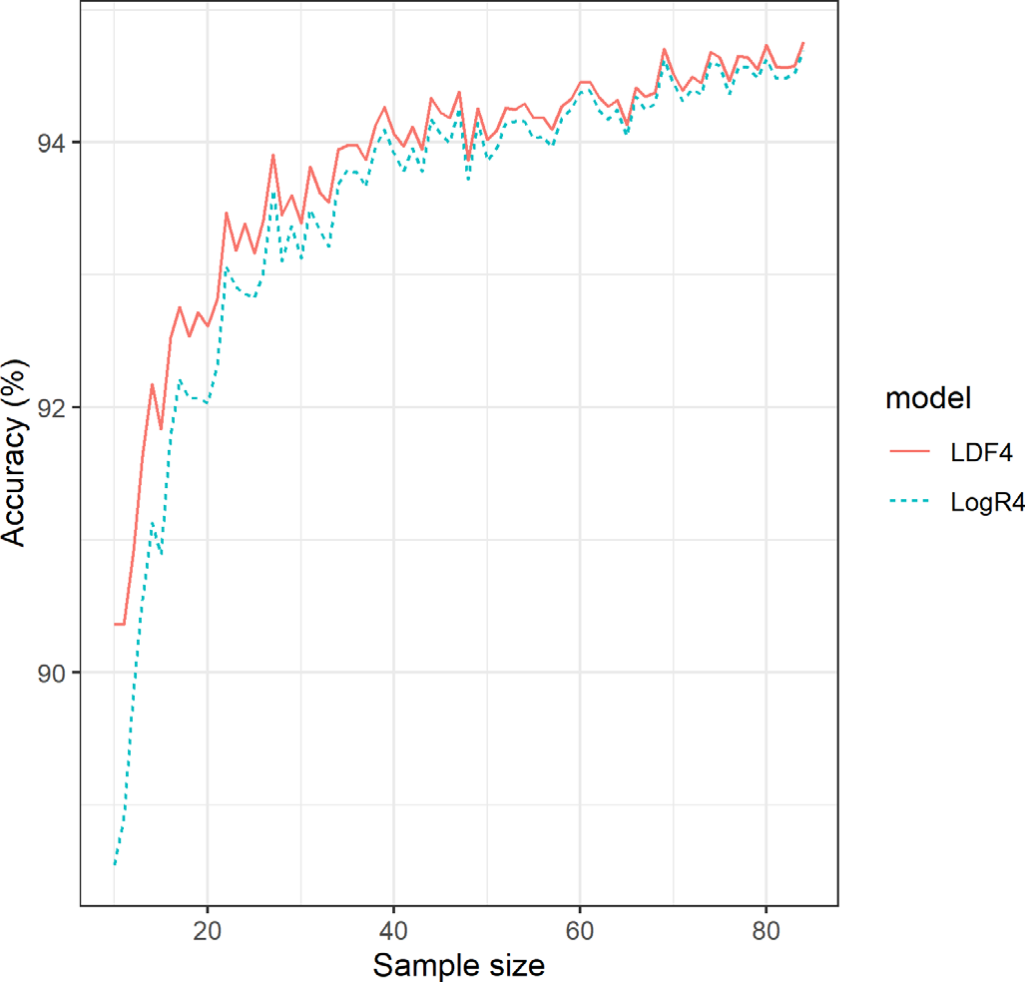
The effect of sample size (*x*‐axis) on the overall accuracy (*y*‐axis) for the LDF4 (solid line) and LogR4 (dotted line) models. [Color figure can be viewed at wileyonlinelibrary.com]

Linear discriminant analysis (LDA) outperformed LogR in overall accuracy across most of the simple and multiple variable equations (Tables 5 and 6) as well as with the *B* index, while LogR performed better with the *Q* index for all simple and multiple variable equations (Table 6). The differences were minor, and often the accuracy rate was only differentiated by the first or second decimal point. By moving the decision cutoff from 0.50 to 0.80 probability, the total classification accuracy increased for all models. The classification rate for female individuals increased for all models, whereas the male classification accuracy decreased for LDF4 and LDF5 (Table 5). Both models had a classification accuracy ranging from 50.00 to 83.33% when only including individuals with classification probabilities lower than 0.80. Female accuracies ranged from 0.00 to 76.47%, and male accuracies ranged from 66.67 to 100%. The male accuracies were higher in all models except for LogR3 (Table 7). Both methods provide similar accuracy rates under varying sample sizes, with LDF4 slightly outperforming LogR4 overall (Fig. 2). The overall mean accuracy across all sample sizes for LDF4 was 93.81% (SD = 0.05498), and for LogR4 it was 93.53% (SD = 0.05980).

**Table 6:** Overall accuracy for individuals with lower than 0.8 classification probability.

Model	Accuracy (%)	Female Accuracy (%)	Male Accuracy (%)
LDF1	67.35	65.38	69.57
LDF2	50.00	0	75.00
LDF3	57.14	53.33	**66.67**
LDF4	**83.33**	50.00	**100**
LDF5	**83.33**	50.00	**100**
LogR1	67.35	65.38	69.57
LogR2	**71.43**	**50.00**	**80.00**
LogR3	**71.43**	**76.47**	63.63
LogR4	71.43	50.00	80.00
LogR5	71.43	50.00	80.00

Bold indicates the model that performed best.

**Table 7:** *B* and *Q* indices for all LDA and LogR models

Equation	*B* index	*Q* index
LDF1	0.8410	0.2973
LDF2	**0.9496**	0.7277
LDF3	0.9071	0.5225
LDF4	**0.9559**	0.7228
LDF5	**0.9559**	0.7222
LogR1	**0.8412**	**0.3038**
LogR2	0.9495	**0.7396**
LogR3	0.9071	**0.5231**
LogR4	0.9533	**0.7435**
LogR5	0.9532	**0.7436**

Bold indicates the model that performed best.

## Discussion

Sexual dimorphism in most skeletal elements occurs on a gradient with an area of considerable overlap between males and females, which is the main source of prediction inaccuracy in any sex estimation study. Methods relying on morphology of traits generally divide them into five categories for sex estimation (female, probable female, indeterminate, probable male, male) (21). These categories can partially account for the overlapping expression of traits between the sexes, which cannot be perfectly dichotomized. Both males and females can show intermediate expression of metric and discrete traits (3), and sexual dimorphism can increase or decrease based on acquired nutrition, hormone levels, activity levels, and age (36, 37). Intrasexual variability among skeletal traits can heavily influence the accuracy of a method (3, 12), and there currently exists no skeletal trait, or combination of traits, that can discriminate perfectly between the sexes. In this study, the probabilities obtained from both methods show that the individuals within the sample follow the entire spectrum from a probability close to 1 (e.g., individual MB5; 0.9997 female) to a probability close to 0.50 (e.g., individual MB16; 0.5463 female), that is, chance alone. The considerable overlap in the skeletal traits of males and females needs to be accounted for when reporting sex estimation results.

When LDA models for sex estimation are built from reference populations, they are often presented in publications as an equation accompanied by a cutoff point that discriminates between males and females depending on what side of the cutoff point the discriminant score lands (1, 11, 38). Ideally, the prediction will be associated with a posterior probability value; however, when using equations from DFA models, the calculation of posterior probabilities for new cases requires the data from the reference sample (which are rarely shared) used to develop the model. In the absence of posterior probabilities, the prediction is necessarily treated as a dichotomous decision, and this can lead to misclassifications. When individuals with a classification probability lower than 0.8 are excluded, the number of misclassifications decreases and the accuracy increases in all models, with increases in accuracy ranging from 0.89 to 12.38% (Table 5). We also showed that the classification accuracy for individuals whose classification probability is lower than 0.80, ranges between 50% and 83%, which is unacceptable for both forensics and osteoarcheology (Table 7). LDA may perform better on smaller sample sizes and when assumptions are met (13, 39); often, however, these two methods will provide similar results (5, 6, 39, 40). In our study, LDA slightly outperformed LogR in overall accuracy and the *B* index for most models, while all LogR models slightly outperformed LDA models in the *Q* index. Ultimately, however, there exists very little difference between the performance of LDA and LogR on a number of indicators and sample sizes.

We recommend the use of LogR due to the flexibility of the method. Similar recommendations have also previously been made for LogR and probit (2, 5, 24). The performances may vary when additional assumptions are violated, and for different samples. Tallman and colleagues (2) found both probit and LogR to outperform DFA in modern Thai individuals, and Walker (5) found LogR to produce a lower sex bias than LDA. LogR also outperformed DFA in the Athens collection (6), while the predictions accuracies in this study were consistent between the two methods. Other discriminant alternatives to LDA are quadratic discriminant analysis (QDA) and nearest neighbor discriminant analysis (NNDA), both of which have fewer assumptions than LDA and have previously seen success in sex estimation (41). While the use of LDA can be theoretically justified when the assumptions are not violated, LogR is more appropriate for predicting a small number of groups, and to develop models for sex estimation that include traits scored on a discrete scale due to the absence of distribution assumptions for the predictor variables (2, 3, 5). Additionally, if certain assumptions are not met, the reliability of a method may be called into question by a court, for example, the Daubert Standard (42). In this study, the LogR analysis may be considered more reliable since its assumptions were met, whereas the assumptions for LDA were not.

Previous studies have provided methods to calculate alternative cutoff points to improve prediction accuracies. Papaioannou and colleagues (9) provide cutoff points for single variable measurements based on 0.80, 0.90, and 0.95 posterior probability values. Hora and Sládek (17) provided software to calculate additional cutoffs from single variable measurements with a custom posterior probability threshold. For models with multiple predictor variables, Giles and Elliot (16) calculated sectioning points indicating confidence of classification at a 0.95 level, assuming a normal distribution of each group’s discriminant scores. We have built upon these methods to provide additional cutoff points at a customizable probability (here, we used classification probabilities of 0.80, 0.90, and 0.95) for both simple and multiple variable equations using bootstrapping, eliminating the assumption of normality. If LDA is being used, we urge authors creating reference equations to share their data so that future users can calculate the posterior probabilities on their unknown cases. If this is not possible, additional cutoff points should be provided.

The development of software that can be used to estimate sex and calculate posterior probabilities should make the publication of equations and cutoff points obsolete, yet this is a practice that seems to persist. Software for metric sex estimation includes FORDISC (43), mainly for North America, and CADOES (44) and SeuPF (45), which are based on Portuguese populations. As the geographic coverage of such software increases, it will allow for more reliable sex estimations with quantifiable uncertainty, and the publication of dichotomy‐imposing equations should become a thing of the past.

## Conclusion

Sex estimation is an important part of the biological profile in skeletal analysis. It is an imperfect science and must be presented as such. There is considerable overlap in the skeletal traits of females and males because sexual dimorphism is a continuum, not a dichotomy. The uncertainty of sex estimation due to the overlap between the sexes should be better represented in the statistical models that are published, to allow future users to properly quantify the reliability of the estimates.

We recommend using logistic regression over discriminant function analysis due to its flexibility and the ability for future users to directly acquire a probability value which they then can use to obtain an informed prediction. If the latter is used, we suggest that the authors of the reference equations provide any of the following information: all measurements and known sex data; posterior probabilities for all individuals in the reference sample; or, at very least, additional cutoff points (PMarks) based on posterior probabilities of 0.80, 0.90, and/or 0.95 for each sex.

## Supporting information


**Table S1.** Data for all individuals included in the study.
